# Bacteriophages as Potential Treatment for Urinary Tract Infections

**DOI:** 10.3389/fmicb.2016.00465

**Published:** 2016-04-11

**Authors:** Wilbert Sybesma, Reinhard Zbinden, Nino Chanishvili, Mzia Kutateladze, Archil Chkhotua, Aleksandre Ujmajuridze, Ulrich Mehnert, Thomas M. Kessler

**Affiliations:** ^1^Neuro-Urology, Spinal Cord Injury Center and Research, University of Zürich, Balgrist University HospitalZürich, Switzerland; ^2^Institute of Medical Microbiology, University of ZürichZürich, Switzerland; ^3^The Eliava Institute of Bacteriophage, Microbiology, and VirologyTbilisi, Georgia; ^4^Tsulukidze National Center of UrologyTbilisi, Georgia

**Keywords:** bacteriophages, antibiotics, urinary tract infection, bacteriophage adaptation, antibiotic resistance

## Abstract

**Background:** Urinary tract infections (UTIs) are among the most prevalent microbial diseases and their financial burden on society is substantial. The continuing increase of antibiotic resistance worldwide is alarming so that well-tolerated, highly effective therapeutic alternatives are urgently needed.

**Objective:** To investigate the effect of bacteriophages on *Escherichia coli* and *Klebsiella pneumoniae* strains isolated from the urine of patients suffering from UTIs.

**Material and methods:** Forty-one *E. coli* and 9 *K. pneumoniae* strains, isolated from the urine of patients suffering from UTIs, were tested *in vitro* for their susceptibility toward bacteriophages. The bacteriophages originated from either commercially available bacteriophage cocktails registered in Georgia or from the bacteriophage collection of the George Eliava Institute of Bacteriophage, Microbiology and Virology. *In vitro* screening of bacterial strains was performed by use of the spot-test method. The experiments were implemented three times by different groups of scientists.

**Results:** The lytic activity of the commercial bacteriophage cocktails on the 41 *E. coli* strains varied between 66% (Pyo bacteriophage) and 93% (Enko bacteriophage). After bacteriophage adaptation of the Pyo bacteriophage cocktail, its lytic activity was increased from 66 to 93% and only one *E. coli* strain remained resistant. One bacteriophage of the Eliava collection could lyse all 9 *K. pneumoniae* strains.

**Conclusions:** Based on the high lytic activity and the potential of resistance optimization by direct adaption of bacteriophages as reported in this study, and in view of the continuing increase of antibiotic resistance worldwide, bacteriophage therapy is a promising treatment option for UTIs highly warranting randomized controlled trials.

## Introduction

Urinary tract infections (UTIs) are among the most prevalent bacterial infections and their financial burden for the society is substantial. In the USA alone, UTIs are responsible for over 7 million physician visits annually (Foxman, 2002) and approximately 15% of all community-prescribed antibiotics are used for UTIs resulting in an annual cost in excess of 1 billion US dollars (Mazzulli, 2002). UTIs account for more than 100’000 hospital admissions annually in the USA (Foxman, 2002), most often for pyelonephritis. These infections are held responsible for at least 40% of all hospital-acquired infections, and the majority of cases are catheter-associated (Rüden et al., 1997). The wide and often uncritical use of antibiotics contributes to the global increase in resistant strains and the present state of microbial resistance is alarming (Carlet et al., 2011; Verbeken et al., 2014). Thus, there is an urgent need for effective, well-tolerated therapeutic alternatives.

After the discovery of bacteriophages by d’Hérelle in 1917, the use of bacteriophage therapy was proposed in the 1920s and has a long and colorful history. At present, bacteriophage therapy is a registered medicine in East European countries like Georgia, Armenia, Ukraine, and Russia, and well accepted by physicians and patients. Several researchers (Abedon et al., 2011; Chanishvili, 2012) have reviewed numerous examples of successfully applied bacteriophage therapy for a diverse area of medical specializations, including dermatology, surgery, wound treatment, intestinal infections, ophthalmology, gynecology and urology. In the Western world bacteriophages have been approved as food processing aid for the decontamination of food (e.g., ListShield^TM^). However, the use of bacteriophages as treatment against selected bacterial infectious diseases has not yet been approved by regulatory authorities and a regulatory framework is still missing (Pirnay et al., 2011, 2015). Nevertheless, the interest from Western clinicians to collaborate with specialists in bacteriophage therapy is rising, as is illustrated by the growing number of scientific papers in peer reviewed journals, for instance regarding the identification of bacteriophages against the O104:H4 *E. coli* outbreak in Germany, (Merabishvili et al., 2012) and the treatment of *P. aeruginosa* and *Staphylococcus aureus* infections in burn wound patients (Merabishvili et al., 2013). In addition, a number of recent reviews have addressed the safety of bacteriophage therapy (Kutter et al., 2010; Pirnay et al., 2015).

Technically, bacteriophage therapy involves the application of bacteriophages that, upon encounter with specific pathogenic bacteria, can infect and kill the pathogenic bacteria. Bacteriophages can specifically dock on bacteria (host species), introduce their DNA, multiply by using the host cell machinery bacteria, and subsequently lyse the bacteria with release of virion progeny that can re-initiate the cycle. In this way, bacteriophages are unique among antibacterial agents in their ability to increase their numbers in the presence of specific bacterial targets.

Bacteriophages might be rather appropriate means to treat patients suffering from UTIs: compared to last generation of antibiotics, bacteriophages are relatively cheap and the treatment can be done under the control of medical personnel by using a catheter for intravesical instillation several times a day. In addition, bacteriophage therapy for UTI treatment will be applied locally and does not involve a systemic treatment. It is also likely that the absence of physical and metabolic barriers in this kind of treatment increases the potential efficacy and reduces the occurrence of potential adverse events. Thus, in view of the high prevalence of UTIs, the continuing increase of antibiotic resistance worldwide, and the renewed interest to apply bacteriophage therapy in the Western world, we investigated the effect of bacteriophages on *E. coli* and *K. pneumoniae* strains isolated from the urine of patients suffering from UTIs.

## Materials and Methods

### Information on Bacterial Strains and Patients

Between February 2012 and January 2013, 41 *E. coli* and 9 *K. pneumoniae* strains were isolated from urinary cultures of 50 patients (22 women and 28 men) suffering from UTIs defined according to the European Association of Urology Guidelines on Neuro-Urology (Groen et al., 2016). Urine samples were collected by sterile catheterization at the Balgrist University Hospital Zürich and sent within 16 h to the microbiological laboratory of the University of Zürich, Switzerland. None of the patients received antibiotic treatment within 4 weeks before urinary culture. The mean age of the 50 patients was 55 ± 18 years. The causes of lower urinary tract dysfunction were spinal cord injury in 38 (76%), multiple sclerosis in 8 (16%) and spinal stenosis in 4 (4%) patients. Of the 50 patients, 34 (68%) relied on aseptic intermittent self-catheterization, 12 (24%) on an indwelling (transurethral *n* = 4, suprapubic *n* = 8) catheter and 4 (16%) voided spontaneously.

The urine samples were cultivated on sheep blood agar (COS, bioMérieux, Marcy l’Etoile, France), on sheep blood agar with colistin and nalidixic acid (CNA, bioMérieux) and on the chromogenic agar (Uriselect 4, Bio-Rad, Marnes – la Coquette, France). The isolated bacteria were identified according to standard procedures. The susceptibility testing was performed according to the EUCAST Version 3.1, 2013 (www.eucast.org). The strains were stored at -70°C in skimmed milk. Before shipping to Georgia, the isolated strains were sub-cultured and transferred to Amies transport agar (Copan, Brescia, Italy).

### Bacteriophages

Four commercial bacteriophage preparations registered in Georgia, Pyo, Intesti, Ses, and Enko bacteriophages, along with 29 *E. coli* and 10 *K. pneumoniae* bacteriophages from the bacteriophage collection of the George Eliava Institute of Bacteriophage, Microbiology and Virology, were used in a bacterial cell lysis screening assay. In order to eliminate effects of batch to batch variations, the individual bacteriophage preparations used for the screening came from the same production batch. The bacteriophages composing the four commercial bacteriophage preparations were grown on a set of bacterial strains, and underwent titration by Appelmans method (Appelmans, 1921) on each individual strain previously used for multiplication of the bacteriophages. In agreement with current regulation the average titer of the mixture is 10^-4^–10^-5^ for at least 48 h (Appelmans method), which corresponds to titers of 10^7^–10^9^ pfu/mL.

The individual bacteriophages have been selected on basis of their capability to eliminate currently prevailing pathogenic microorganisms in Georgia, including *E. coli*, *P. aeruginosa, Staphylococcus* spp., *Shigella* spp., *Salmonella* spp., *Enterococcus* spp., and *Proteus* spp. More information of the composition and origin of the four bacteriophage preparations can be found in earlier work on Pyo and Intesti (Abedon et al., 2011) and Ses and Enko (Fitzgerald-Hughes et al., 2014).

The 29 *E. coli* bacteriophages were freshly prepared and originally isolated for their potential as antimicrobial agents for therapeutical applications from a broad range of different sources during the period 2000–2012, like sewage waters, agricultural farm isolates (Khurtsia et al., 2001) and clinical isolates (Ghudumidze et al., 2012).

The 10 *K. pneumoniae* bacteriophages (**Table 3**) were isolated between 2002 and 2012 from sewage samples (Karumidze et al., 2013) with the aim to treat patients suffering from UTIs and prostatitis using clinical *K. pneumonia*e strains or *K. oxytoca* strains as hosts. All bacteriophages used in this study were lytic bacteriophages, as was concluded on basis of a combination of analyses and observations, including plaque morphology and clarity of lytic zones on the lawn of bacterial agar, host range, time to infection, time of latent period, and concentration of progeny (Clokie and Kropinski, 2009).

### Screening

Screening of bacterial strains for susceptibility to bacteriophages was performed by using a spot test method (Craigie and Yen, 1938) and then modified by different authors and adjusted for different species (Felix and Callow, 1943; Felix, 1956). Bacterial cultures were grown overnight (18–24 h at 37°C) to receive a concentration of 10^9^ colony forming units (cfu) mL^-1^ and diluted 10 times up to a final concentration of 10^8^ cfu mL^-1^. 100 μL of the diluted culture was transferred into 2–3 mL of previously melted and cooled soft agar (0.7% Brain-Heart agar), vortexed for 30–60 s and poured on the top of solidified and previously dried Petri dishes containing 2% of Brain-Heart agar. After solidification of the top agar (30–60 min) at room temperature, 10 μL of bacteriophage suspension with a titer of 10^7^ plaques forming units (pfu) mL^-1^ was placed on top of the soft agar using an automatic pipette. The plates were let to dry for 20–30 min and then incubated overnight (18–24 h) at 37°C. Next, the results were visually evaluated.

### Primary Outcome Measure

**FIGURE 1:**
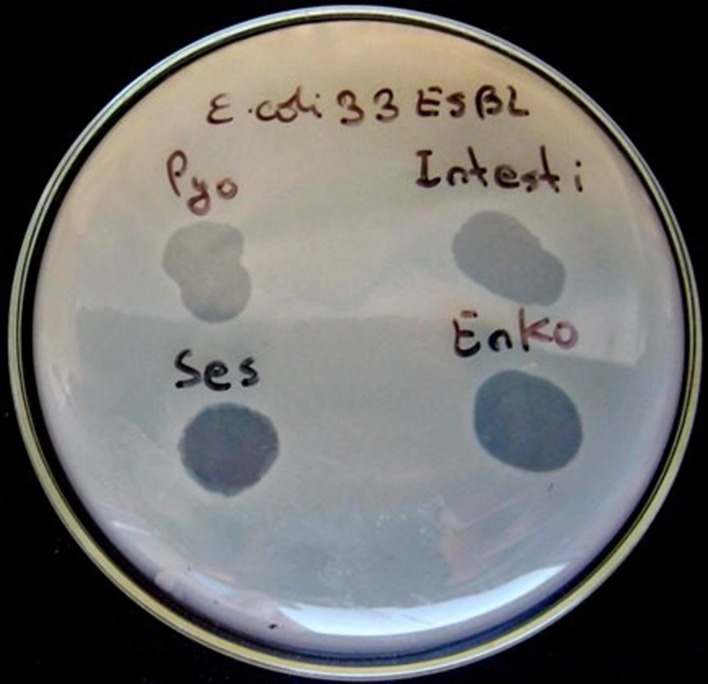
**Plaque morphology of *Escherichia coli* strain #33.** The figure shows overgrown (partial) lysis (OL) in case of Pyo and Intesti bacteriophages and confluent (complete) lysis (CL) in case of Ses and Enko bacteriophages. All these results are positive.

**FIGURE 2:**
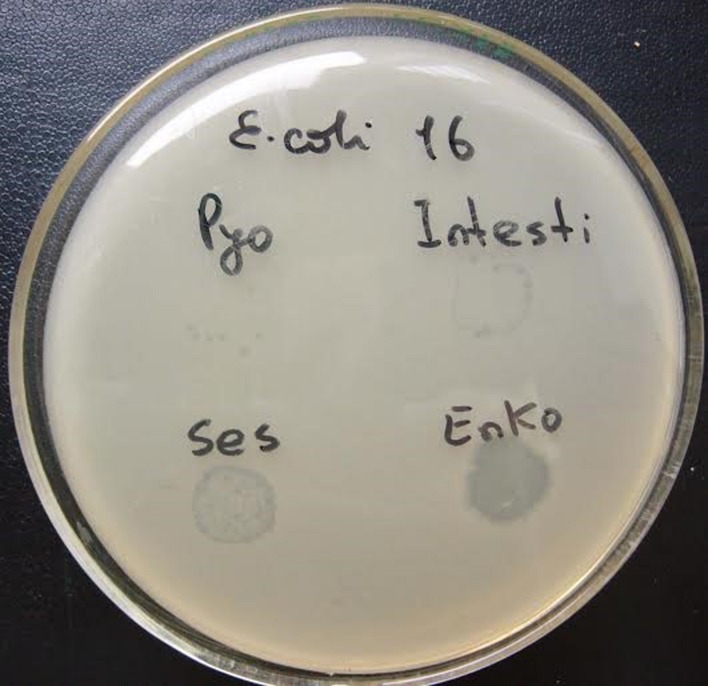
**Plaque morphology of *E. coli* strain #16.** The figure shows individual plaques (IP) in case of Pyo and Intesti bacteriophages and two degrees of overgrown (partial) lysis (OL) in case of Ses and Enko bacteriophages. All these results are positive.

The primary outcome measure was the assessment of lytic activity of the bacteriophages on the bacteria, as could be identified by the plaque morphology in a double-layered plaque assay using *E. coli* or *K*. *pneumoniae* strains. A bacterial lawn inoculated with a range of bacteriophages gives different visual patterns, similar to a bacteriophage typing pattern and depending on the effect of the bacteriophage-host strain interaction. The clear zones usually differ by their intensity and structure (**Figures 1** and **2**). The positive reactions were classified as “CL” – confluent lysis, i.e., a full clear zone on the well-developed lawn of bacterial culture; “SCL” – semi-confluent lysis, i.e., a not fully cleared bacterial lawn; “OL” – overgrown lysis, i.e., single bacterial colonies that overgrew a complete clear zone; “IP” – individual clear or opaque plaques, which look like multiple small clear zones on the bacterial lawn within a drop contour, previously assigned by d’Hérelle as “tv” from “*taches vierges*” (Clokie and Kropinski, 2009; Chanishvili, 2012). The negative reactions were classified as “R” – resistant, i.e., no lysis.

### Extension and Optimisation of the Bacteriophage Preparation Host-Range (Adaptation)

Expansion of the range of lytic activity of the Pyo bacteriophages cocktail was achieved by selection of mutant bacteriophages with extended host-range (h-mutants) and by increasing their titers in the bacteriophage preparation. For this purpose the bacteriophages were propagated in a suspension containing the bacterial cultures of choice with low or no susceptibility to these bacteriophages. The initial steps of adaptation technique are similar to Appelmans’ method for titration in liquid media (Appelmans, 1921). The adapted bacteriophages were used for the repeated screening.

## Results

The antibiogram of the 41 *E. coli* and 9 *K. pneumoniae* strains shows the ESBL production capability of the strains and their sensitivity or resistance for each of seven tested antibiotics, **Table 1**. In summary, the strain susceptibility varies between sensitive for all antibiotics to resistant for at least six of the seven tested antibiotics. The lytic (killing) activity of the commercial bacteriophage cocktails on the 41 *E. coli* strains varied between 66% (27 strains sensitive for Pyo bacteriophages) and 93% (38 strains sensitive for Enko bacteriophages), **Table 2**. From the 29 *E. coli* bacteriophages from the Eliava collection, the highest lytic activity came from the bacteriophages vB_*E. coli* _4t, vB_*E. coli* _4s, and vB_*E. coli* _Pic with 42, 39, and 24% coverage, respectively. Three of the *E. coli* strains (*E. coli* 5, *E. coli* 29, and *E. coli* 35 ESBL) showed complete resistance to all bacteriophage from any of the 4 commercial bacteriophage cocktails and 29 *E. coli* bacteriophages from the Eliava collection.

**Table 1:** Antibiogram of 41 *Escherichia coli* and 9 *Klebsiella pneumoniae* strains isolated from the urine of patients suffering from UTIs.

	Antibiotic resistance profile							
	AM	AMC	CXM	SXT	NOR	CIP	NF	ESBL
***E. coli*** **strain number**
3,9,10,13,17,19,21,22, 23,25,27,28,38,39,40	S	S	S	S	S	S	S	N
12	S	S	S	S	S	S	R	N
11,30	S	S	S	S	R	R	S	N
29,36	R	S	S	S	S	S	S	N
14,24	R	S	S	S	R	R	S	N
8	R	S	S	S	I	S	S	N
37	R	S	S	R	S	S	S	N
7,16,18,20	R	S	S	R	R	R	S	N
1,4	R	S	R	S	S	S	S	Y
5	R	S	R	S	S	S	S	N
2,31	R	S	R	S	R	R	S	Y
15	R	S	R	S	R	R	S	N
35	R	S	R	S	R	R	R	Y
41	R	S	R	R	R	R	S	Y
6	R	R	S	R	R	R	S	N
26	R	R	S	I	R	R	S	N
32	R	R	R	R	S	S	S	Y
33,34	R	R	R	R	R	R	S	Y
****K. pneumoniae**** **strain number**
43	R	S	S	S	S	S	S	Y
46,50	R	S	S	S	S	S	S	N
42	R	S	S	S	S	S	ND	N
48	R	S	S	R	S	S	S	N
49	R	S	R	R	S	S	ND	Y
47	R	S	R	R	R	R	S	Y
44	R	S	R	R	I	I	R	N
45	R	R	R	R	R	R	ND	Y

*Strains were isolated from 22 different women and 28 different men with age between 25 and 80. Numbers 1–41 are *E. coli* strains with at least 18 different antibiotic resistance profiles. Numbers 42–50 are *K. pneumoniae* strains with at least seven different antibiotic resistance profiles. AM, ampicillin; AMC, amoxicillin/clavulanic acid; CXM, cefuroxime; SXT, sulfamethoxazole/trimethoprim or co-trimoxazole; NOR, norfloxacin; CIP, ciprofloxacin; NF, nitrofurantoin; ESBL, extended-spectrum-beta-lactamase; Y, yes (present); N, no (absent); R, resistant; S, sensitive; I, intermediate; ND, not done. Notwithstanding the occurrence of several identical antibiograms, all strains have different bacteriophage resistance/sensitivity profiles, except strains 14 and 24 (compare with **Tables 2** and **3**).*

**Table 2:** Results of spot tests on 41 *E. coli* strains using commercial bacteriophage cocktails.

Strain number	Commercial bacteriophage cocktails				
	Pyo	
	Before adaptation	After adaptation	Intesti	Ses	Enko
39	CL	CL	CL	OL	OL
7	IP	IP	IP	OL	OL
20	IP	OL	IP	OL	OL
6,16	IP	SCL	IP	OL	OL
9	OL	CL	OL	OL	OL
1	OL	CL	OL	SCL	OL
23	OL	OL	CL	OL	OL
12,21,33	OL	OL	OL	CL	CL
31	OL	OL	OL	IP	SCL
4,8,14,24,26,30,34	OL	OL	OL	OL	OL
40	OL	OL	OL	SCL	OL
28	OL	OL	SCL	CL	CL
27,32	OL	OL	SCL	SCL	SCL
17	OL	OL	SCL	OL	OL
38	OL	SCL	CL	SCL	SCL
19	OL	SCL	OL	OL	OL
3	R	CL	OL	IP	OL
18,36,37	R	CL	OL	OL	OL
25	R	CL	R	R	OL
15	R	CL	R	SCL	OL
22	R	CL	R	SCL	SCL
2	R	CL	SCL	SCL	CL
5	R	IP	R	R	R
10	R	IP	SCL	SCL	SCL
11	R	R	R	OL	SCL
35	R	R	R	R	R
41	R	R	SCL	SCL	CL
29	R	SCL	R	R	R
13	SCL	SCL	SCL	CL	CL
Lytic activity	65.9%	92.7%	82.9%	90.2%	92.7%

*CL, confluent lysis; SCL, semi-confluent lysis; OL, overgrown lysis; IP, individual plaques; R, resistant. Twenty-nine unique bacteriophage resistance/sensitivity profiles can be distinguished.*

However, after adaptation of the Pyo bacteriophages the lytic activity could be increased from 27 to 38 sensitive strains (66 to 93%, **Table 2**), including two of the three previously mentioned resistance strains. In total, all but one *E. coli* strain could be lysed by one of the four commercial bacteriophage preparations and only strain *E. coli* 35 ESBL remained resistant, even after the adaptation of the Pyo bacteriophage preparation.

Regarding *K. pneumoniae* strains (**Table 3**), the best result was shown by bacteriophage v_B–KpS10 that could lyse all nine strains.

**Table 3:** Results of spot tests on 9 *K. pneumoniae* strains using laboratory bacteriophages from the Eliava collection.

	Bacteriophages									
Strain number	vB_KlpR1	vB_KloxR2	vB_KlpR3	vB_KlpR4	vB_KlpR5	vB_KlpR6	vB_KlpR7	vB_KlpR8	v_BR KpM 9	v_BRKpS 10
45,47	R	R	OL	OL	R	R	R	R	OL	OL
42	R	R	R	R	R	CL	R	CL	OL	OL
48	R	R	R	R	R	R	R	OL	R	SCL
50	R	R	R	R	R	R	R	R	OL	OL
43,44,46	R	R	R	R	R	R	R	R	R	OL
49	R	R	R	R	R	R	R	R	SCL	CL
Lytic activity	0%	0%	22%	22%	0%	11%	0%	22%	56%	100%

*CL, confluent lysis; SCL, semi-confluent lysis; OL, overgrown lysis; IP, individual plaques; R, resistant. Six unique bacteriophage resistance/sensitivity profiles can be distinguished.*

Bacteriophage and antibiotic susceptibility/resistance did not correlate. For example, *E. coli* strain #33 was resistant to all tested antibiotics except nitrofurantoin (NF; **Table 1**), while the same strain was susceptible to all tested bacteriophages (**Table 2**, **Figure 1**). On the contrary, *E. coli* strain #35 was resistant to all tested bacteriophages (**Table 2**), but showed susceptibility to two antibiotics, i.e., amoxicillin (AMC) and sulfamethoxazole (SXT; **Table 1**).

## Discussion

The *in vitro* lytic activity of bacteriophages has already been demonstrated in several studies (Chanishvili, 2012) but data on the application of bacteriophages for treating UTIs are scarce (Perepanova et al., 1957; Arshba and Bagdoeva, 1965; Kolomeitsev et al., 1966; Danilova, 1996; Letkiewicz et al., 2010; Khawaldeh et al., 2011; Chanishvili, 2012). In the present study we showed for the first time the lytic activity of commercially existing bacteriophage cocktails on 41 *E. coli strains*, isolated from urinary cultures of patients suffering from UTIs. The applied spot test demonstrated the results of the bacteriophage–bacteria interactions: All reactions assigned as CL, SCL, OL, and IP were positive, which means that the bacteriophages lysed the bacteria, although with different degrees of effectiveness. In case of CL the bacteria could not develop bacteriophage resistance during the incubation period on the plate (18 h), indicating that this kind of bacteriophage–bacterium interaction can be considered as the most effective one. The other spot morphologies reflected less lytic activity, which could partially be caused by the different concentrations of specific bacteriophages in the commercial cocktails. Interestingly, our results show a similar order of lytic activity for the four commercial bacteriophages (before adaptation) as reported in an earlier bacteriophage sensitivity test on *E. coli* strains (Fitzgerald-Hughes et al., 2014). Furthermore, in the present study, we have shown that, as is common in the development of bacteriophages, the impact and activity of the bacteriophage cocktails can be increased in adaptation experiments, as illustrated by the change from IP into OL, or even CL (**Table 2**). The commercial bacteriophage preparations, after the adaptation experiments of the Pyo bacteriophage cocktail in successive series of adaptation, showed lytic activity on 40 of the 41 *E. coli* strains. *K. pneumoniae* bacteriophages from the Eliava collection could lyse all of the 9 *K. pneumoniae* strains tested. Besides adaptation, the efficacy of bacteriophage cocktails can also be increased by addition of new bacteriophages that show lytic activity toward the targeted clinical bacterial isolate.

Since the mode of action of antimicrobial drugs like antibiotics differs considerably from bacteriophages, resistance mechanisms are consequently different, as can be concluded by comparing **Table 1** with **Tables 2** and **3**. Furthermore, these data also indicate that all but two bacterial strains with a similar antibiotic resistance profile have different bacteriophage resistance profiles, which indicates a broader (genetic) variability among the isolates than could have been expected on the basis of the antibiotic resistance profiles only. Although beyond the scope of this study, detailed genetic characterisation could indicate the effect of bacteriophages on specific serotypes or genotypes of *E. coli* and *K. pneumonia* and determine the spectrum of the (adapted) bacteriophage cocktails vs. the diversity of the strains. Full genome sequencing could further reveal which defense mechanisms by the bacteria and which counter-strategies by the bacteriophages may have been evolved (Samson et al., 2013).

The applied bacteriophage cocktails Pyo, Intesti, Ses, and Enko are allowed by the Georgian medicinal products legislation. In terms of safety, these bacteriophage preparations are already commercially available for more than 50 years and have been used millions of times without relevant incidence of adverse events. In addition, the bacteriophages in these cocktails are all lytic and do not have the ability to integrate and remain in the bacterial genome, contrarily to the temperate bacteriophages that after infection include their DNA into the host genome without making much harm to the host (see Materials and Methods). For a complete overview on quality and safety requirements for sustainable bacteriophage therapy products see (Pirnay et al., 2015).

Notwithstanding the potential of application of bacteriophage therapies in treating patients suffering from UTIs, and its broad acceptance in some East European countries, one major limitation today is the regulatory framework in the Western world (Verbeken et al., 2014; Pirnay et al., 2015), which requires a long and expensive road of assessments and randomized, placebo-controlled, double-blind trials. These studies should also investigate the best routes of administration, duration and dosage of treatment, adverse events, stability and definition of formulations and applicability for acute infections. Furthermore, and in order to accelerate potential regulatory approval for bacteriophage therapy, bacteriophages should not be considered in a similar way as chemical drugs, but, because of their typical mode of action, more like the components of the influenza vaccine, which is re-evaluated and changed yearly and does not require clinical trials for each revision (Pirnay et al., 2011). In addition, it may be appropriate to classify bacteriophages in different ways for different applications. For example as a drug for systemic applications, but as a medical device for more topical applications, as proposed for the treatment of UTIs (Pirnay et al., 2011). Recently, a set-up has been proposed where bacteriophage therapy is coordinated and standardized (in a first instance) by national bacteriophage therapy centers, which operate under the supervision of relevant public health authorities and in interaction with private stakeholders (Pirnay et al., 2015).

## Conclusion

The results of this study have clearly shown the lytic activity of commercial bacteriophage cocktails on *E. coli* and *K. pneumonia* strains isolated from patients suffering from UTIs. In addition, the presented data has shown the potential of bacteriophage adaptation experiments with the aim to increase the lytic activity of the bacteriophage cocktails.

In times of increasing antibiotic resistance worldwide and in combination with the long history of safe use of bacteriophages in East European countries, our findings can be seen as a stimulus to invest in the set-up of randomized, placebo-controlled trials and investigate the efficacy of bacteriophage therapies in the Western world as a common alternative to strictly chemical-based treatment of bacterial infections.

In view of the high prevalence of UTIs, bacteriophages, as natural and self-amplifying antibacterial “drugs,” might offer a non-systemic effective therapeutic option urgently warranting randomized controlled trials.

## Author Contributions

All authors had full access to all of the data in the study and take responsibility for the integrity of the data and the accuracy of data analysis. Study concept and design: WS, TMK. Acquisition, analysis, or interpretation of data: WS, RZ, MK, NC, AC, AU, UM, TMK. Drafting of the manuscript: WS and TMK. Critical revision of the manuscript for important intellectual content: RZ, MK, NC, AC, AU, UM. Data analysis: WS, TMK. Obtained funding: UM, TMK. Study supervision: TMK. All authors read and approved the final version to be published.

## Conflict of Interest Statement

The authors declare that the research was conducted in the absence of any commercial or financial relationships that could be construed as a potential conflict of interest.

## Abbreviations

AM: ampicillin
AMC: amoxicillin/clavulanic acid
CIP: ciprofloxacin
CL: confluent lysis
CXM: cefuroxime
*E. coli*: *Escherichia coli*
ESBL: extended spectrum beta lactamase
EUCAST: European Committee on Antimicrobial Susceptibility Testing
F: female
I: intermediate
IP: individual clear or opaque plaques
*K. oxytoca*: *Klebsiella oxytoca*
*K. pneumonia*: *Klebsiella pneumoniae*
M: male
n: no (absent)
ND: not done
NF: nitrofurantoin
NOR: norfloxacin
OL: overgrown lysis
*P. aeruginosa*: *Pseudomonas aeruginosa*
R: resistant
S: sensitive
SCL: semi-confluent lysis
SXT: sulfamethoxazole/trimethoprim or co-trimoxazole
tv: taches vierges
UTIs: urinary tract infections
y: yes (present).
